# The effect of crystalloid *versus* medium molecular weight colloid solution on post-operative nausea and vomiting after ambulatory gynecological surgery - a prospective randomized trial

**DOI:** 10.1186/1471-2253-12-15

**Published:** 2012-07-31

**Authors:** Ivan Hayes, Raza Rathore, Kingsley Enohumah, Edgar Mocanu, Deepak Kumar, Conan McCaul

**Affiliations:** 1 Department of Anaesthesia, The Rotunda Hospital Dublin, Parnell Square, Dublin 1, Ireland; 2 Royal College of Surgeons in Ireland, Dublin, Ireland; 3 School of Medicine and Medical Sciences, University College Dublin, Dublin, Ireland; 4 Mater Misericordiae University Hospital, Dublin, Ireland

**Keywords:** Fluid therapy, Colloid, Crystalloid solutions, Nausea, Vomiting

## Abstract

**Background:**

Intravenous fluid is recommended in international guidelines to improve patient post-operative symptoms, particularly nausea and vomiting. The optimum fluid regimen has not been established. This prospective, randomized, blinded study was designed to determine if administration of equivolumes of a colloid (hydroxyethyl starch 130/0.4) reduced post operative nausea and vomiting in healthy volunteers undergoing ambulatory gynecologic laparoscopy surgery compared to a crystalloid solution (Hartmann’s Solution).

**Methods:**

120 patients were randomized to receive intravenous colloid (N = 60) or crystalloid (N = 60) intra-operatively. The volume of fluid administered was calculated at 1.5 ml.kg^-1^ per hour of fasting. Patients were interviewed to assess nausea, vomiting, anti-emetic use, dizziness, sore throat, headache and subjective general well being at 30 minutes and 2, 24 and 48 hours post operatively. Pulmonary function testing was performed on a subgroup.

**Results:**

At 2 hours the proportion of patients experiencing nausea (38.2 % *vs* 17.9%, P = 0.03) and the mean nausea score were increased in the colloid compared to crystalloid group respectively (1.49 ± 0.3 *vs* 0.68 ± 0.2, P = 0.028). The incidence of vomiting and anti-emetic usage was low and did not differ between the groups. Sore throat, dizziness, headache and general well being were not different between the groups. A comparable reduction on post-operative FVC and FEV-1 and PEFR was observed in both groups.

**Conclusions:**

Intra-operative administration of colloid increased the incidence of early postoperative nausea and has no advantage over crystalloid for symptom control after gynaecological laparoscopic surgery.

## Background

The incidence of post operative nausea and vomiting (PONV) following ambulatory surgery is 40%–60% and ambulatory gynecological patients are at particularly high risk. PONV continues to occur despite pharmacological prophylaxis in high risk groups. [1-6] PONV has the potential to cause delays in meeting discharge criteria both from the recovery room to ward and from the day ward to home. [7-9] PONV causes patient discomfort and can result in unanticipated overnight hospital admission which leads to increased economic costs. PONV control is a strong patient priority and there is a strong association between PONV and patient dissatisfaction with their anesthesia care. [10-12] The routine use of anti-emetics remains controversial as their efficacy is limited in patients with low risk profiles. [13] In these circumstances, pharmacologic prophylaxis increases the risk of adverse drug effects for the patient in addition to increasing overall costs of care [13].

‘Rehydration’ is a recommended strategy to reduce PONV but have been subject to a relatively small number of studies in ambulatory anesthesia and there is no consensus as to which fluid or volume is optimal. [14-18] Studies suggest that intravenous crystalloid administration in healthy patients reduces the incidence of nausea, vomiting and antiemetic use after gynecological laparoscopy and other ambulatory procedures. [19-22] The anti-emetic efficacy of intravenous crystalloid appears to be dose related. Lower volumes do not have a significant effect and large volumes (30–40 ml/kg) are necessary to establish benefit. [19,23,24] Intravenous crystalloid has a short intravascular half life, its expected duration of action is short and solutions with longer durations of action e.g. colloid would be anticipated to have greater benefit. [25] We chose to study the effect of intravenous colloid solution in PONV and hypothesized that intravenous colloid would have a greater reduction on PONV than an equivalent volume of crystalloid solution.

## Methods

Following approval by the Institutional Research Ethics committee (Rotunda Hospital, Dublin, Ireland), ASA I-II female patients, aged 18–45 years who were scheduled to undergo elective gynecological laparoscopy were invited to participate in the study. The study took place in the operating theatres, recovery room and day ward of the Rotunda Hospital, a tertiary level university teaching hospital. Exclusion criteria included age <18 or >45 years, cardio-respiratory disease, excessive intra-operative blood loss, BMI >30 and relevant drug allergy to medication used in the protocol. Informed written consent was obtained from all patients. Patients were randomized using a table of computer generated numbers each of which was linked to a sealed envelope that contained the allocation to one of two groups. The crystalloid group (CRL) received Compound Sodium Lactate at a volume of 1.5 ml.kg^-1^.hr^-1^ of fasting time administered as an intra-operative bolus. Compound Sodium Lactate (Baxter Health Care, Thetford, UK) contains sodium 131 mmol litre^−1^, potassium 5 mmol litre^−1^, calcium 2 mmol litre^−1^, chloride 111 mmol litre^−1^ and lactate 29 mmol litre^−1^. The colloid (CLD) group received 6% Hydroxy Ethyl Starch 130/0.4 in 0.9% saline (Voluven®; Fresenius Kabi, Bad Homburg, Germany) at a volume of 1.5 ml.kg^-1^.hr^-1^ of fasting time as an intra-operative bolus. To ensure blinding of both the patient and the investigator, all fluid administration was initiated after induction of anesthesia and completed prior to transfer to PACU. No further fluid was administered after this time. Neither the patient nor the investigator was aware of group assignment.

All patients received general anesthesia. Intravenous access was obtained after local anesthetic infiltration of the skin. Anesthesia was induced with fentanyl 1.5 mcg.kg^-1^, Propofol 2–3 mg.kg^-1^ and Atracurium 0.35 mg.kg^-1^ to facilitate airway control. The lungs were ventilated via a supraglottic airway (LMA Supreme™^TM^ size 4) with tidal volume (Vt) 4–6 ml.kg^-1^, maximum airway pressure (P max) 30 cm H_2_O and a positive end expiratory pressure (PEEP) of 5 cm H_2_O. The respiratory rate was adjusted to maintain isocapnia. Anaesthesia was maintained with 1 MAC of Sevoflurane in a mixture of O_2_ and air to achieve a FiO_2_ of 0.5. Muscle relaxation was antagonized with neostigmine 2.5 mg and glycopyrrolate 0.5 mg at the end of surgery. Before discontinuation of anesthesia, each patient received rectal Diclofenac 100 mg and Paracetamol 1000 mg i.v*.* Prophylactic anti-emetics were not administered.

Postoperative care was standardized. In PACU, patients reporting pain were given 25 mcg of Fentanyl *i.v.* as required to achieve a pain score of 4 or less. On the ward Pethidine 50 mg intramuscularly or simple oral analgesics (Paracetamol/Codeine 500-1000 mg/8-16 mg were administered on a 3 and 6 hourly PRN basis as required respectively. Rescue anti-emetics were available in the form of ondansetron 4 mg *i.v.* in PACU and cyclizine 50 mg on the ward every 8 hours on a demand basis. In the event of failure of cyclizine, prochlorperazine 12.5 mg IM was available 8 hourly. Patients were prescribed oral Paracetamol/Codeine 500–1000 mg/8–16 mg every 6 hours and Diclofenac 50 mg every 8 hours to take at home if required.

The primary endpoint was the presence of nausea at 2 hours post-operatively. Nausea was assessed using a standard verbal rating scale (VRS 0 = no nausea, 10 = worst imaginable). [20] Secondary endpoints included vomiting, rescue antiemetic use, dizziness, sore throat and headache. Dry retching was considered to be vomiting for the purposes of the study. Symptoms were recorded as present or absent at each time point i.e. cumulative data were not reported. Anti-emetics were offered by PACU nursing staff to patients if they reported nausea when clinically assessed using a modified Aldrete score. [26] Pre and post-operative pulmonary function tests were performed in a subset of 30 patients following consultation with the institutional ethics committee after development of post-operative pulmonary edema in a study patient. Pulmonary function was assessed at the bedside using Viasys Microlab Spirometer (Viasys Healthcare, Warwick, United Kingdom) Patients were assessed by a single data collector (RR) at 30 mins, 2 hours, 24 hours and 48 hours postoperatively. The 24 and 48 hour assessments were carried out by telephone interview.

Statistics were performed using Sigma Stat, (Jandel Scientific). Categorical data were analyzed using the χ 2 test or the Fisher's exact test as appropriate. Continuous data were analyzed with the Student-*t* test or repeated measures analysis of variance as appropriate. Normally distributed data are presented as means and standard deviation and data that was not normally distributed is presented as medians and interquartile range. Categorical data is presented as proportions and percentages.

Sample size was calculated as follows: To detect a clinically significant difference of 25% reduction in mean nausea scores based on a baseline score of 40 mm, we calculated a sample size of 60 patients per group (a = 0.05, β = 0.2).

## Results

There were no baseline differences between the groups in terms of age, weight, BMI, risk factors for PONV, fasting duration or anaesthetic technique (Table 1).

**Table 1:** Patient characteristics

	**Crystalloid**	**Colloid**	**P value**
Age (yr)	35.1 (4.3)	35.6 (5.3)	0.55
Female (%)	100	100	1.0
Weight (kg)	63.7 (59.0–72.0)	64.0 (54.7–70.0)	0.61
BMI (kg.m^2^)	23.7 (21.6–25.4)	22.9 ( 20.9–25.1)	0.34
Duration of Fast (Hr)	13.0 ( 11.0–17.0)	14.0 (12.0–15.7)	0.38
**PONV Risk Factors (N)**			
1	22	21	0.94
2	32	30	0.93
3	1	3	0.6
4	0	0	1.0
**Procedure**			
Laparoscopy – Diagnostic (N)	39	38	0.88
Laparoscopy – Proceed (N)	17	17	0.88
Estimated Fluid Deficit (L)	1.37 (0.34)	1.36 (0.38)	0.88
Volume of Fluid Received (ml/kg)	21.0 (4.7)	21.6 (5.5)	0.58
Duration of Anesthesia (min)	40.0 (30.0–50.0)	40.0 (30.0–45.0)	0.14
Hypotensive Episodes requiring Treatment (N)	11	8	0.62
Intraperitoneal CO_2_ (L)	9.1 (7.0–15.6)	10.5 (8.1–17.5)	0.33
Propofol ( mg.kg^-1^)	2.6 (2.4–3.0)	2.6 (2.4–2.9)	0.7
Atracurium (mg.kg^-1^)	0.37 (0.34–0.43)	0.37 ( 0.35–0.41)	0.9

Continuous data are presented as mean, standard deviation and median, interquartile range. Categorical data are presented as Number and percentage.

**Figure 1:**
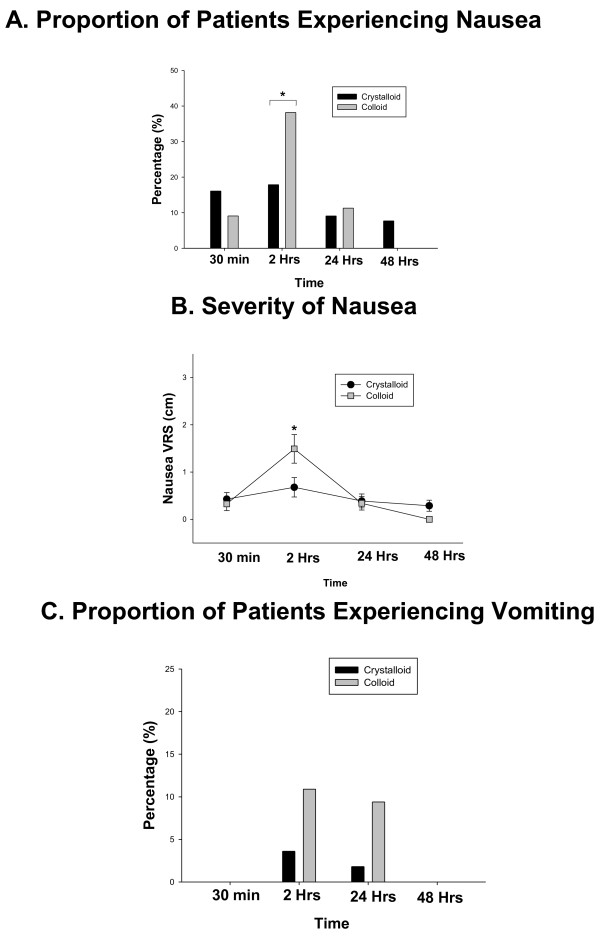
**Panel A: Data are percentage.** *Indicates *P* < 0.05. Panel **B:** Verbal Analogue Score of Nausea. (0 = No Nausea 10 = Worst Possible) Data are non parametric and presented as mean and standard error of the mean. * Indicates P = 0.028. Panel **C:** Data are percentage.

**Figure 2:**
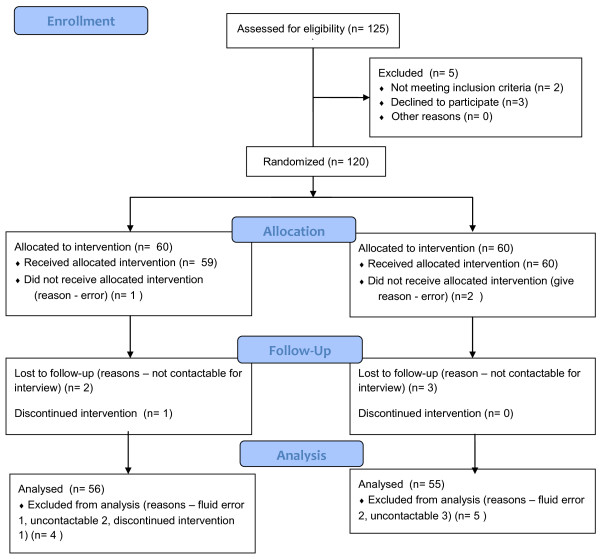
Consort flow diagram.

**Figure 3:**
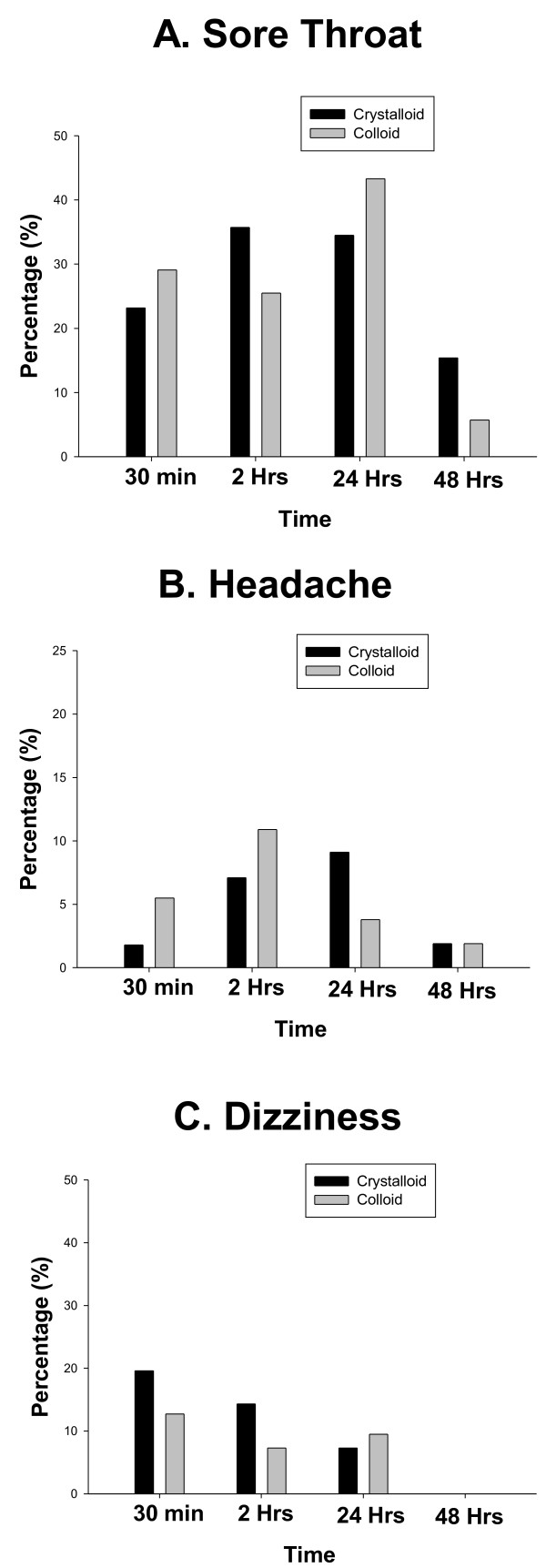
All data are percentage.

**Figure 4:**
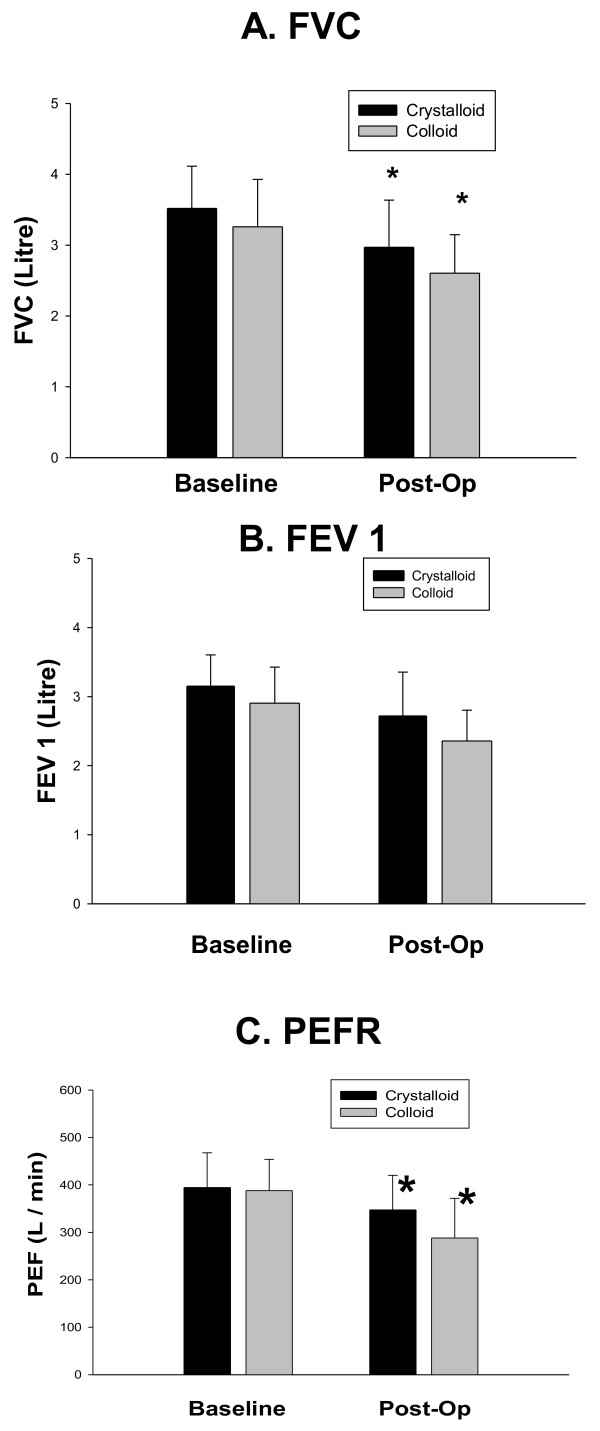
**Panel A: Data are non parametric and presented as mean and standard error of the mean.** * Indicates *P* < 0.05 for post operative crystalloid and colloid vs. baseline (P=0.013, 0.016 respectively). Panel **B:** Data are non parametric and presented as mean and standard error of the mean. * Indicates *P* < 0.05 for post operative crystalloid and colloid vs. baseline (P=0.025, 0.012 respectively). Panel **C:** Data are non parametric and presented as mean and standard error of the mean. * Indicates *P* < 0.05 for post operative crystalloid and colloid vs. baseline (P=0.001, 0.004 respectively).

At 2 hours the proportion of the patients experiencing nausea (38.2 *vs* 17.9%, P = 0.03) (Figure 2, Panel A) and the mean nausea score (1.49 ± 0.3 *vs* 0.68 ± 0.2, P = 0.028) (Figure 2, Panel B) and were increased in the colloid group compared to crystalloid respectively. There was no difference between the groups in these outcomes at any other time. The incidence of vomiting (Figure 1, Panel C) and anti-emetic usage (Table 2) was low and did not differ between the groups. Post-operative analgesic use was not different between the groups. (Table 2) Sore throat, dizziness, headache and general well being were not different between the groups post-operatively (Figure 3 Panels A-C). A comparable reduction on post-operative FVC and FEV-1 and PEFR was observed in both groups (Figure 4 Panels A-C).

**Table 2:** Post-operative medication

**Opioids**	**Crystalloid**	**Colloid**	**P value**
Fentanyl (mcg)	25 (0.0–100)	25 (0.0–75.0)	0.52
Pethidine (mg)	10.3 (23.7)	4.9 (16.2)	0.17
**Rescue Anti-emetics**			
Number of Patients Requiring Rescue			
Anti-emetics N(%)	9 (16.9)	5 (5.3)	0.37
**Time of Anti-emetic administration. N (%)**			
0–30 min	2 (3.6)	3 (5.3)	0.98
30 min–2 hrs	5 (9.1)	2 (3.5)	0.42
2–24 hrs	3 (5.7)	0 (0.0)	0.22

Continuous data are presented as mean, standard deviation and median, interquartile range. Categorical data are presented as Number and percentage. Note – One patient required anti-emetic more than one occasion.

One patient was withdrawn from the colloid group after developing high airway pressures in the operating room. In PACU a chest x-ray showed pulmonary edema which was treated with diuretics and oxygen. She was well at follow up.

## Discussion

Multiple previous prospective randomized clinical trials have demonstrated a reduction in post-operative nausea and vomiting associated with intravenous crystalloid administration which is dose related. [19-22,24,27,28] Intravenous fluid administration has also been shown to improve multiple other common post-operative symptoms and to shorten time to achieving discharge criteria. [21,23] Recent consensus guidelines by the ASA and ASPAN recommend peri-operative rehydration to reduce baseline risk of PONV but no specific fluid formulation or volume or rate of administration have been advocated. [16,17] The present study aimed to determine whether a colloidal solution may have any advantage over crystalloid in magnitude or duration of effect on PONV. We did not confirm any such benefit and in fact demonstrated an increase in early nausea in patients receiving the colloid solution. The observed nausea was mild and did not affect general well being. Secondary outcomes – the incidence of headache, sore throat and dizziness were not improved by colloid administration.

There have been a small number of related studies in diverse patient populations to date and none in ambulatory care specifically. The results of these studies have been conflicting. Haentjens, in a prospective randomised trial investigated the effect of hydroxyethyl starch 130/0.4 or saline in a mixed surgical population undergoing non-ambulatory surgery and showed no difference between the groups in nausea and vomiting. [29] The volumes of experimental fluids were similar to those used in our study but were administered over a 24 hour period whereas in our study they were administered intra-operatively – a considerably shorter time period of greater relevance to the ambulatory surgical population. Moretti investigated the effect of 6% hetastarch in either balanced salt (HS-NS) or lactated Ringer’s solution (HS-BS) with lactated Ringer’s in an older population undergoing major intracavity surgery. [30] Fluid administration was algorithmically determined and PONV was more common in patients receiving Ringer’s lactate and clinical evidence suggested that significant interstitial oedema developed in this group. The authors speculated that the reduction in PONV in the colloid group was due to less bowel edema. It is perhaps unsurprising that tissue edema occurred in the crystalloid group as these patients received a mean volume of 5946 ml compared to 1301 ml and 1448 ml in the HS-NS and HS-BS groups respectively. In a study of healthy non-anaesthetised volunteers, subjects receiving 50 ml/kg of sodium chloride reported abdominal discomfort more frequently than those who received an equivalent volume of lactated Ringer’s solution, indicating a possible difference in the tendency to cause bowel edema between these solutions. [31] Wilkes studied 6% hetastarch in either Hartmann’s solution or 0.9% Sodium Chloride in elderly patients undergoing open surgery and showed improved gastric perfusion and a trend toward less vomiting in the former group, suggesting that the electrolyte composition of the colloidal solution has some influence on its emetogenic potential. [32] In a study of female patients undergoing elective laparoscopic cholecystectomy, Chaudhary did not demonstrate any differences on PONV between groups receiving 12 ml.kg^-1^ of Ringers lactate or 12 ml.kg^-1^ 4.5% Hydroxyethyl starch prior to induction of anaesthesia. This study was notable for an extremely high incidence of PONV. [33] The colloid intervention group also received a subsequent infusion of Ringers lactate and final fluid volumes were not reported. In a study of patients undergoing cholecystectomy, Turkistani administered 10 ml.kg^-1^ of one of three colloidal study solutions of low, medium and high molecular weight respectively. [34] The study was underpowered to detect a difference between colloid groups but showed a distinct trend towards greater PONV with increasing molecular weight.

In the studies which have been carried out to date on the effects of intravenous fluids on PONV after gynaecological laparoscopy, the specific mechanism of action of fluid has not been determined. Intravenous fluids have not been found to be beneficial in thyroidectomy and therapeutic abortion suggesting that the effect may be specific to laparoscopic surgery. [35,36] Speculative mechanisms of PONV after laparoscopy include a reduction in gut mucosal blood flow that is known to occur during pneumoperitoneum and anaesthesia. [37] Mesenteric hypoperfusion in turn may lead to release of 5-hydroxytryptamine which is a potent trigger of nausea and vomiting. High volume intravenous fluids have also been shown to reduce post-operative pain and could potentially reduce opioid consumption, both of which can be emetogenic. We chose to study hydroxyethyl starch 130/0.4 which is a medium molecular weight starch in a balanced electrolyte solution designed to minimize tissue accumulation and coagulation dysfunction while retaining the hemodynamic efficacy of conventional starches. [38] We anticipated that the colloidal study solution would have a more marked and sustained effect than crystalloid on restoration of intravascular volume and consequently reduce intestinal mucosal ischemia and subsequent PONV. [39] The findings of the study were opposite to that expected and a greater proportion of the patients who received colloid were nauseated. We speculate that this observation is explicable by a relative increase in blood viscosity that has been previously reported with hydroxyethyl starches that may paradoxically impair microcirculatory perfusion. The observation that highest incidence of nausea occurred at 2 hours post-operatively in both groups and occurred more commonly in patients who had received intravenous fentanyl in PACU, suggests that the mechanism of nausea in this study population was at least in part opioid induced, which is not known to be ameliorated by intravenous fluid administration.

Our study has a number of limitations. *First*, there was no untreated control group and a single dose of colloid was evaluated, therefore beneficial effects of the solution at different doses cannot be excluded. *Second*, blinding is incomplete as the anaesthetist who administered the fluids was not blinded. *Third*, the study was conducted in patients without cardiorespiratory disease and use of such volumes of rapidly administered intravenous fluids cannot be recommended to all patients. *Fourth*, the study was underpowered to demonstrate statistical differences for secondary outcomes, particularly pulmonary function which showed clear trends towards greater impairment in the colloid group. *Fifth*, following discharge from PACU oral intake was not restricted or measured. *Last*, prophylactic anti-emetics were not administered, limiting the ability of clinicians to extrapolate of our findings to patients who receive such interventions.

## Conclusion

In conclusion, we were unable to demonstrate a benefit of colloid over crystalloid in PONV after ambulatory gynecologic surgery and cannot recommend it at the investigated dose for this purpose. 

## Competing interests

None of the authors have any financial or non-financial competing interests.

## Authors’ contributions

IH designed the study, analyzed the data and drafted the manuscript. RR collected data and was involved in drafting the manuscript. KE collected data, was involved in its analysis and manuscript drafting. EM was involved in drafting the manuscript. DK collected data. CMC designed the study, co-ordinated it, analyzed the data and drafted the manuscript. All authors saw and approved the final manuscript.

## Funding

Internal Department only.

## Pre-publication history

The pre-publication history for this paper can be accessed here:

http://www.biomedcentral.com/1471-2253/12/15/prepub
